# Prevalence of HIV-related stigma manifestations and their contributing factors among people living with HIV in Sweden – a nationwide study

**DOI:** 10.1186/s12889-024-18852-9

**Published:** 2024-05-20

**Authors:** Lena Nilsson Schönnesson, Marie Dahlberg, Maria Reinius, Galit Zeluf-Andersson, Anna-Mia Ekström, Lars E. Eriksson

**Affiliations:** 1 Department of Global Public Health, Karolinska Institutet, Stockholm, Sweden; 2 Department of Learning, Informatics, Management and Ethics, Karolinska Institutet, Stockholm, Sweden; 3 Department of Learning, Informatics, Management and Ethics, Medical Management Centre, Karolinska Institutet, Stockholm, Sweden; 4 Department of Infectious Diseases, South General Hospital/Venhälsan, Stockholm, Sweden; 5 Department of Neurobiology, Care Sciences and Society, Karolinska Institutet, Huddinge, Sweden; 6 School of Health and Psychological Sciences, City, University of London, London, UK; 7 Medical Unit Infectious Diseases, Karolinska University Hospital, Huddinge, Sweden

**Keywords:** HIV, Stigma, People living with HIV, Sweden

## Abstract

**Background:**

With access to  antiretroviral therapy (ART) HIV infection is a chronic manageable condition and non-sexually transmissible. Yet, many people living with HIV still testify about experiencing HIV-related stigma and discrimination. It is well-documented that HIV-related stigma and discrimination continue to be critical barriers to prevention, treatment, care and quality of life. From an individual stigma-reduction intervention perspective, it is essential to identify individual and interpersonal factors associated with HIV-related stigma manifestations. To address this issue and to expand the literature, the aim of this study was to assess the prevalence of HIV-related stigma manifestations and their associated factors among a diverse sample of people living with HIV in Sweden.

**Method:**

Data from 1 096 participants were derived from a nationally representative, anonymous cross-sectional survey ”Living with HIV in Sweden”. HIV-related stigma manifestations were assessed using the validated Swedish 12-item HIV Stigma Scale encompassing four HIV-related stigma manifestations: personalised stigma, concerns with public attitudes towards people living with HIV, concerns with sharing HIV status, and internalized stigma. Variables potentially associated with the HIV-related stigma manifestations were divided into four categories: demographic characteristics, clinical HIV factors, distress and ART adherence, and available emotional HIV-related support. Four multivariable hierarchical linear regression analyses were employed to explore the associations between multiple contributors and HIV-related stigma manifestations.

**Results:**

The most dominating stigma feature was anticipation of HIV-related stigma. It was manifested in high scores on concerns with sharing HIV status reported by 78% of the participants and high scores on concerns about public attitudes towards people living with HIV reported by 54% of the participants. High scores on personalised stigma and internalized stigma were reported by around one third of the participants respectively. Between 23 and 31% of the variance of the four reported HIV-related stigma manifestations were explained mainly by the same pattern of associated factors including female gender, shorter time since HIV diagnosis, feelings of hopelessness, non-sharing HIV status, and lack of available emotional HIV-related support.

**Conclusion:**

The most dominating stigma feature was anticipation of stigma. Female gender, shorter time since HIV diagnosis, feelings of hopelessness, non-sharing HIV status, and lack of available emotional HIV-related support constituted potential vulnerability factors of the four HIV-related stigma manifestations. Our findings highlight the vital necessity to support people living with HIV to increase their resilience to stigma in its different forms. Exploring associated factors of HIV-related stigma manifestations may give an indication of what circumstances may increase the risk of stigma burden and factors amenable to targeted interventions. As individual stigma-reductions interventions cannot be performed isolated from HIV-related stigma and discrimination in society, a key challenge is to intensify anti-stigma interventions also on the societal level.

## Background

With access to antiretroviral therapy (ART), HIV infection is a chronic manageable condition with a long-life expectancy [1]. ART is a combination of medications that interfere with one or more steps of the virus’ lifecycle. The aim of ART is to reduce the levels of HIV, as the virus destroys T-helper (CD4-positive) cells of the immune system. If the viral load is low enough – also called viral suppression – tests won’t be able to detect HIV in the blood and further sexual HIV transmission is being prevented, also known as U = U (Undetectable = Untransmittable). Optimal adherence to ART is central to achieving viral suppression. In spite of successful medical achievements, HIV infection, like many other chronic or sexually transmissible health conditions is surrounded by stigma [2, 3]. The term stigma originated in ancient Greece where it referred to a mark made on the skin by hot iron or by cutting/pricking as a symbol of infamy [4].

Within the HIV context, stigma refers to social stigma, which “can only be understood in relation to broader notions of power and domination” ([5], p16). It originates within social contexts and social processes [5–7] and is grounded in socially constructed divisions of “us” as compared to a “them” [8]. HIV-related stigma, which is of global concern, includes negative attitudes and beliefs about people living with HIV. It is well documented that living with HIV is supremely stigmatized [9]. HIV-related stigma is driven by several factors such as fear of infection, the perception of preventability, the near-universal lethality of its complications if untreated, and the association of the infection transmission with socially marginalized groups such as people who inject drugs, sex workers, and men who have sex with men [10]. These drivers may put the individual at risk for being perceived by others as deviant, undesirable or unworthy and treated as if they were invisible, non-existent, or socially dead [6]. When stigma is acted upon, the result can be rejection and discrimination [11].

People living with HIV are confronted with structural and community stigma [12]. Structural stigma refers to “societal-level conditions, cultural norms, and institutional practices that constrain the opportunities, resources, and wellbeing for stigmatized populations” ([12], p. 2). Structural stigma can be identified through state and national laws, regulations, and policies. Another example of structural stigma is stigma within health care facilities. It is widely documented that stigmatization within health care is “ranging from outright denial of care, provision of sub-standard care, physical and verbal abuse, to more subtle forms, such as making certain people wait longer or passing their care off to junior colleagues” ([13], p. 1). Community stigma on the other hand refers to the awareness of the stigmatizing beliefs and attitudes about people living with HIV that exist in the community [14]. For example, within the gay community HIV-positive men who have sex with men (MSM) perceive a cause of division related to HIV leading to feelings of sexual rejection and discrimination [15].

Earnshaw & Chaudoir emphasize that the societal level of conceptualizations of stigma “should be complemented by individual level conceptualizations of stigma” (p. 1161), as people living with HIV are well aware of societal negative attitudes and prejudices towards their HIV status as well as discriminatory behaviours [16]. This knowledge is experienced through 3 stigma mechanism enacted, anticipated and internalized stigma. Enacted stigma refers to the degree to which people living with HIV believe they have actually experienced prejudice and discrimination from others as a result of one’s HIV-status as manifested in avoidance, rejection or isolation. Anticipated stigma refers to the degree to which people living with HIV expect to be treated negatively due to one’s HIV-positive status. Internalized stigma refers to the degree to which people living with HIV accept and apply societal negative attitudes to the self as manifested in shame, self-blame, embarrassment, and low self-worth. These mechanisms represent “the psychological responses to the knowledge that they, themselves, may have violated social mores and may be subject to other people’s negative treatment” ([16], p. 1162). The HIV Stigma Framework presented by Earnshaw & Chaudoir [16] suggests that these stigma mechanisms could have different outcomes related to health and wellbeing for people living with HIV. Moreover, to fully understand the mechanisms and effects of HIV-related stigma, it is imperative to acknowledge how multiple identities intersect (e.g. race, class, gender, sexual orientation) and how they impact stigma experiences [5, 17, 18]. Research shows that people living with HIV who are multiply stigmatized experience worse HIV treatment outcomes [19].

Research clearly indicates that overall HIV-related stigma, intersectional stigma and structural stigma are key barriers to HIV testing and access to HIV care [12, 20] and often plays a role in suboptimal ART adherence [21] as well as disengagement from HIV care [5, 22]. Moreover, HIV-related stigma is a chronic stressor [23] and it is well-documented that it has a negative impact on mental health, physical health, health-related behaviours, and overall health-related quality of life [24–29]. Extensive cross-sectional and longitudinal research has found that internalized HIV stigma is one of the most important dimensions associated with adverse HIV outcomes such as ART non-adherence, higher viral load and poor mental health outcomes [30–32]. People living with HIV consistently report a poorer emotional wellbeing when compared to the general population and people with other chronic illnesses [33].

Stigma and discrimination often constitute a much more distressing factor for people living with HIV than the illness itself [34] and there is a strong commitment worldwide to eliminate HIV-related stigma [11]. UNAIDS underscores that by 2025 “less than 10% of people living with HIV and key populations experience stigma and discrimination” [35]. From a stigma-reduction intervention perspective, it is important to assess the prevalence of different manifestations of HIV-related stigma [36] and associations between individual characteristics and HIV-related stigma. Studies have shown that gender, age, sexual orientation, socio-economic status, education, living in rural areas, being single, racism, maladaptive coping, depression, anxiety, lack of locus of control, lack of social support, and time since HIV diagnosis are significantly associated with high HIV-related stigma [17, 24, 25, 27, 34, 37–41].

There is limited quantitative research on the correlates of different types of HIV-related stigma (except internalized stigma) [42]. It is thus of concern to acknowledge this issue, as associated factors may differ amongst HIV-related stigma manifestations, which otherwise may be undetected by an overall HIV stigma measure [23]. To address this issue and to expand the literature, the aim of this study was to assess the prevalence of HIV-related stigma manifestations and their contributing factors among a diverse sample of people living with HIV in Sweden.

### Theoretical framework

The theoretical basis of this study is the HIV stigma framework [16], which focuses on stigma mechanisms (enacted, anticipated and internalized). Earnshaw & Chaudoir [16] hypothesize that these stigma mechanisms could have different affective, behavioral and physical outcomes related to health and well-being among people living with HIV. The present study assesses potential associated factors of HIV-related stigma manifestations without claiming directionality in line with the crossectional design of the the study.

Based on prior research literature, HIV community and clinical experiences, the associated factors were divided into three layers. The first one relates to demographic characteristics and clinical HIV factors, the second one to distress and ART adherence and the final one to available emotional HIV-related support including loved ones, friends and health care support.

### The Swedish context

Sweden is a low-endemic country with an HIV prevalence of about 0.08%. It is estimated that approximately 8 200 people are currently living with a known HIV diagnosis and are in health care contact. Of these, 98% are on antiviral therapy out of which 95% have undetectable viral loads. About one third of people living with HIV are women. Migrants, who were diagnosed with HIV either before or after arrival in Sweden make up approximately 65% of people living with HIV in Sweden. Approximately 40% of people living with HIV in Sweden are 50 years or older. One hundred and forty persons are under the age of 18 years of whom the majority has been living with HIV since birth or very early age. The most common transmission route is heterosexual sexual contacts followed by sexual contacts between men having sex with men and people injecting drugs [43]. Sweden was in 2016 the first country in the world to reach the UNAIDS 90-90-90 target [1]. According to the Swedish Communicable Diseases Act, testing for, clinic visits for and treatment of HIV are free. People living with HIV are also entitled to free psychosocial support. HIV infection is covered by the Swedish Discrimination Act under the issue of discrimination on the grounds of disability.

According to Swedish law, all diagnosed HIV infections are mandatory registered in the Swedish national HIV case reporting system, using an identifying code. Contact tracing is mandatory according to the Swedish Communicable Disease Act. People living with HIV are also obligated to inform sexual partners of their HIV status and to use condoms for vaginal, anal and oral sex. However, the Public Health Agency of Sweden has modified the implementation of the law. HIV physicians can since 2013 exempt patients from sharing their HIV status and using condoms provided they are durably virally suppressed. However, despite the modification the law still exists.

## Methods

### Study design

Data for the present study was drawn from the cross-sectional nation-wide anonymous survey, *Living with HIV in Sweden*, executed in 2014 [44]. The aim of the survey was to explore quality of life and its associated factors in a representative sample of people living with HIV in Sweden. The survey was developed in partnership with representatives from non-governmental HIV support organisations including HIV peer organisations, clinicians and scientists working in the HIV field. The survey was tested by people living with HIV followed by minor adjustment. It resulted in 77 items covering a wide range of issues of potential relevance for the life situation of people living with HIV in a national context of almost complete (98%) coverage of ART and 95% viral suppression [1]. All study procedures were approved by the Regional Ethical Board, Stockholm (DNR 2013/1552-31/4).

#### Participants and procedures

Eligibility criteria included individuals aged 18 years or older, who had been diagnosed with HIV more than six months prior to the study. Enrolment of participants was made from 15 infectious disease outpatient clinics in Sweden and two needle and syringe exchange clinics in Stockholm that together provided ART and follow-ups for 75% of the known adult population living with HIV in Sweden at the time of data collection. A representative sample of 1 100, equalling 17% of the individuals with a known HIV diagnosis (*N* = 6 469) at the time of data collection, was attained. Of these 1 100 individuals, four were excluded (three stated an age under 18 and one had known their HIV status for less than six months) leaving data from 1 096 participants to be included in the present analysis. Recruitment and data collection procedures are described in further detail elsewhere [44, 45].

Information about the study was spread through HIV peer organisations, the survey website and posters at the participating healthcare units. In connection to an ordinary planned clinic visit, potential participants were consecutively informed about the study by the appointed contact person, emphasising voluntary participation and that a response to the anonymous pen-paper-questionnaire indicated consent. Participants could complete the questionnaire in a confidential setting at their outpatient clinic, put the questionnaire in a sealed envelope, and drop it in a locked box (or complete it at home and mail it in a pre-stamped envelope to the research team). The survey was available in Swedish, English, French, Spanish, Russian, Thai, Somali, Amharic, Arabic, and Tigrinya and oral telephone interpretation was available for those with reduced literacy to ensure representation of migrant sub-populations in Sweden. Most participants (82%) responded to the survey in Swedish. No incentive was given.

#### Measures

##### Dependent variable

HIV-related stigma manifestations were measured using the validated Swedish 12-item HIV Stigma Scale [46]. It is a further development of the validated Swedish 40-item HIV Stigma Scale [47], which in turn originates in Berger et al’s HIV Stigma Scale [24]. The Swedish 12-item HIV Stigma Scale [46] measures four stigma manifestations. Personalised stigma refers to experience of discrimination of one’s HIV being known by other people. Anticipated stigma encompasses two aspects: concern with public attitudes towards HIV-positive people (worries about general attitudes and treatment of people living with HIV) and concerns with sharing HIV status (controlling information and concerns related to sharing one’s HIV status). In the original Berger HIV Stigma Scale [24] the latter manifestation was labelled Disclosure concerns. However, as the term disclosure is stigmatizing implying that the person has something to hide/be guilty about [48], the term concerns of sharing HIV status is preferred. The fourth manifestation is internalized stigma (negative feelings towards oneself because of one’s HIV infection).

Each stigma manifestation consists of three items (Table 1) individually rated on a four-point Likert-scale ranging from strongly disagree (1) to strongly agree (4). Item responses for each dimension scale were added up to yield a score from 3 to 12, with mean scores of *≥* 7.5 indicating high levels of perceived HIV-related stigma [46]. Cronbach’s α for the personalised stigma, concerns about public attitudes towards people living with HIV, concerns about sharing HIV status and internalized stigma were 0.89, 0.82, 0.84, and 0.80 respectively.

**Table 1:** Items making up each HIV-related stigma manifestation

*Personalised stigma*
Some people avoid touching me once they know I have HIV.
People I care about stopped calling after learning I have HIV.
I have lost friends by telling them I have HIV.
*Concerns with sharing HIV status*
Telling someone I have HIV is risky.
I work hard to keep my HIV a secret.
I am very careful who I tell that I have HIV.
*Concerns with public attitudes towards people living with HIV*
People with HIV are treated like outcasts.
Most people believe a person who has HIV is dirty.
Most people are uncomfortable around someone with HIV.
*Internalized stigma*
I feel guilty because I have HIV.
People’s attitudes about HIV make me feel worse about myself.
I feel I’m not as good a person as others because I have HIV.

##### Independent variables

Based on research, HIV community and clinical experiences, potential variables associated with HIV-related stigma manifestations were selected from the *Living with HIV in Sweden* survey. They were divided into four categories: demographic characteristics, clinical HIV factors, distress and ART adherence, and available emotional HIV-related support. The response categories on the independent variables are reported in Tables 2 and 3. All variables were self-reported.

**Table 2:** Descriptive statistics: Demographic characteristics and clinical HIV factors (*N* = 1 096)

Demographic characteristics	Frequency *N* (%)
*Gender*	
Male	762 (69.5)
Female	320 (29.2)
Other/missing	14 (1.3)
*Age*	
Less than 30 years	55 (5.0)
30 to 49 years	534 (48.7)
50 to 64 years	346 (31.6)
65 years or older	98 (8.9)
Missing	63 (5.7)
*Country or region of origin*	
Sweden	600 (54.7)
Europe	87 (7.9)
North America	28 (2.6)
Africa	176 (16.1)
Asia	57 (5.2)
Missing	148 (13.5)
*Residency permit*	
Yes	423 (38.6)
No	22 (2.0)
Missing	651 (59.4)
*Education*	
Less than 1 year	12 (1.1)
1 to 6 years	49 (4.5)
7 to 9 years	146 (13.3)
10 to 12 years	365 (33.3)
More than 12 years	496 (45.3)
Missing	28 (2.6)
*Occupation*	
Working	654 (59.7)
Not on the job market	104 (9.5)
Long-term sick leave/retirement	145 (13.2)
Student	74 (6.8)
Missing	119 (10.9)
*Income*	
* <*5000	109 (10.0)
5000 to 13,999	225 (20.5)
14,000 to 21,999	211 (19.3)
22,000 to 31,999	228 (20.8)
* >*32,000 or more	223 (20.3)
Missing	100 (9.1)
*Residential status*	
Living with partner/family	566 (51.6)
Shared accommodation	81 (7.4)
Living alone	390 (35.6)
Have no home	37 (3.4)
Missing	22 (2.0)
*Sexual orientation*	
Heterosexual	481 (43.9)
Homosexual/gay/MSM	394 (35.9)
Bisexual	66 (6.0)
Queer/Trans	11 (1.0)
Missing	144 (13.1)
*Relationship status*	
In a relationship	436 (39.8)
Not in a relationship	175 (16.0)
Missing	485 (44.3)
*Having children*	
No	594 (54.2)
Yes	483 (44.1)
Missing	19 (1.7)
*Drug use during past 6 months* ^*1*^	
Do not take drugs	868 (79.2)
Non-injecting drug use	106 (9.7)
Injecting drug use	38 (3.5)
Missing	84 (7.7)
**Clinical HIV factors**	
*Route of HIV transmission*	
Through sexual contact with a man	609 (55.6)
Through sexual contact with a woman	162 (14.8)
Through infected needles and syringes	83 (7.6)
Through blood/blood products	36 (3.3)
Through perinatal transmission	13 (1.2)
Other way	70 (6.4)
Missing	123 (11.2)
*Time since HIV diagnosis*	
6 to 12 months	47 (4.3)
1 to 5 years	217 (19.8)
6 to 10 years	287 (26.2)
11 to 20 years	292 (26.6)
>20 years	224 (20.4)
Missing	29 (2.6)
*Self-reported current CD4 count*	
<100	110 (10.0)
100 to 200	71 (6.5)
200 to 350	90 (8.2)
350 to 500	84 (7.7)
* ≥*500	146 (13.3)
Missing	595 (54.3)
*Currently experiencing HIV physical symptoms*	
No	702 (64.0)
Yes	209 (19.1)
Missing	185 (16.9)
*On antiretroviral treatment (ART)*	
Yes	1037 (94.6)
No	42 (3.8)
Missing	17 (1.6)
*Current physical ART side effects*	
No	687 (62.7)
Yes	306 (27.9)
Missing	103 (9.4)
*Concurrent treatment for hepatitis C*	
No	342 (31.2)
Yes	18 (1.6)
Missing	736 (67.2)

**Table 3:** Descriptive statistics: Distress and ART adherence and Available emotional HIV-related support (*N* = 1 096)

**Distress and ART adherence**	**Frequency** ***N* (%)**
**Emotional distress**	
*Feelings of hopelessness*	
Absence	486 (44.3)
Mild	251 (22.9)
Moderate	112 (10.2)
Severe	126 (11.5)
Missing	121 (11.0)
*Taking medications for sleeping problems*	
No	754 (68.8)
Yes	223 (20.3)
Missing	119 (10.9)
*Taking medications for worry/anxiety*	
No	777 (70.9)
Yes	122 (11.1)
Missing	197 (18)
*Taking medications for depression*	
No	771 (70.3)
Yes	125 (11.4)
Missing	200 (18.2)
*Taking medications for neuropsychiatric problems*	
No	788 (71.9)
Yes	38 (3.5)
Missing	270 (24.6)
**Sexual distress**	
*Impact of HIV on sex life*	
Negative	626 (57.1)
Positive	138 (12.6)
No change	166 (15.1)
Missing	166 (15.1)
*Fear of being reported to the authorities by a sex partner*	
No	319 (29.1)
Yes	609 (55.6)
Missing	168 (15.3)
*Fear of transmitting HIV to others*	
No	339 (30.9)
Yes	423 (38.6)
Do not have sex	207 (18.9)
Missing	127 (11.6)
*Wish to have children in the future*	
No	407 (37.1)
Yes, it is very important to me	168 (15.3)
Yes, but it is not so important	99 (9.0)
Yes, but did not know PLWH could have children	42 (3.8)
Do not know	127 (11.6)
It is too late	186 (17.0)
Missing	67 (6.1)
**ART adherence**	
*ART Adherence (number of missing doses in the past week)*	
No doses	847 (77.3)
One or more doses	148 (13.5)
Missing	101 (9.2)
**Available emotional HIV-related support**	**Frequency *N* (%)**
*Support from partner, family, friends and others*	
Always	224 (20.4)
Often	220 (20.1)
Sometimes	172 (15.7)
Rarely	85 (7.8)
Never	71 (6.5)
I have not told anyone about my HIV	191 (17.4)
Missing	133 ()12.1
*Trust in HIV physician*	
Completely	938 (85.6)
Partly	84 (7.7)
No	23 (2.1)
Missing	51 (4.7)
*Trust in HIV nurse*	
Completely	982 (89.6)
Partly	48 (4.4)
No	7 (0.6)
Missing	59 (5.4)
*Trust in HIV counsellor*	
Completely	250 (22.8)
Partly	48 (4.4)
No	48 (4.4)
Missing	750 (68.4)

*Demographic characteristics* included gender, age, country or region of origin, permit for Swedish residency, education level, occupation, income, residential status, sexual orientation, relationship status, having children and drug use including use of pharmaceuticals without prescription during the past 6 months.

*Clinical HIV factors* included route of HIV transmission, time since HIV diagnosis, current CD4 count, currently experiencing HIV physical symptoms, ART status, ART side effects and concurrent treatment for hepatitis C.

*The distress and ART adherence category* included emotional and sexual distress and ART adherence. Two psychological aspects were used to measure *emotional distress.* Hopelessness, reflecting negative expectations concerning oneself and one’s future and feelings of helplessness to change those expectations, was measured by the self-reported Beck Hopelessness Scale [49]. The other aspect referred to having received medication for sleeping problems, worry/anxiety, depression, and or neuropsychiatric problems in the past 6 months.

*Sexual distress* included impact of HIV on sex life, fear of being reported to authorities (County Medical Officer, the police) by a sex partner, fear of transmitting HIV to others, and wish to have children in the future.

*ART adherence* here defined as no missed doses was measured by the question “How many doses of ART did you miss during the past week?”

*Available emotional HIV-related support* included experienced emotional support from partner, family, friends and others and trust in healthcare support resources (HIV physician, HIV nurse, and HIV counsellor respectively).

#### Data analysis

Statistical analyses were performed with IBM SPSS version 28 and included three steps. First, descriptive statistics of the independent variables were calculated. Second, a series of bivariable one-way analyses of variance were conducted to explore the association of each independent variable with each of the four stigma manifestations. The alpha level was set to 0.05. Third, multivariable hierarchical linear regression analysis was performed for each stigma manifestation utilising three forced steps. All independent variables were included as categorical variables. The category having the highest frequency was consistently used as reference category. The responses ‘do not know’ and ‘do not want to answer’, as well as missing responses were all categorized as ‘missing’.

Only variables that had statistically significant bivariable associations with the respective stigma manifestation were included in the corresponding model. Demographic characteristics and clinical HIV factors were added in the first step, the distress and ART adherence in the second step and the available emotional HIV-related support factors in the third and final step. Self-reported sexual orientation, income and relationship status were excluded from the analysis due to multicollinearity (VIF tolerance > 0.4). Sexual orientation correlated with HIV transmission route, income with occupation, and relationship status with residential status.

The α-level for significance was Bonferroni adjusted to 0.0125 since four regression models were calculated [50]. Standardised regression coefficients’ (β_s_) contribution to the regression models were considered weak if β_s_ < 0.2, moderate if β_s_ 0.2–0.5 and strong if β_s_ > 0.5. Effect sizes of R^2^ for the regression models were considered as small if R^2^ 0.02–0.14, medium if R^2^ 0.15–0.35 and large if R^2^ > 0.35 [51].

To manage different degrees of missing data on the stigma manifestations the following strategy was applied. When the score was missing for one of the three items in a stigma manifestation, the missing value was imputed with a random of the individual’s responses of the two remaining items of the given stigma manifestation. When an individual had two missing scores of the three items in a stigma manifestation, the missing values were imputed with the individual’s single response on the remaining item. If all three items of a stigma manifestation had missing values, data were excluded from further analyses.

Data on the personalised stigma manifestation was available for 1 004 (92%) participants, 1 037 (95%) participants for the concern with public attitudes towards people living with HIV manifestation, 1 060 (97%) participants for the concerns with sharing HIV status manifestation, and 1 060 (97%) participants for the internalized stigma manifestation.

## Results

### Descriptive statistics

As shown in Table 2, the vast majority of the participants were of male gender (70%) and the average age of the participants was 47.6 (SD 11.81, range 18 to 82) years. More than half of the participants were born in Sweden. About one half of the participants reported being non-heterosexual. More than one third were in an intimate relationship at time of data collection. The majority had at least some post-high school education and 60% were working.

The highest stigma score was observed in concerns with sharing HIV (Mean: 9.18, SD: 2.80), followed by concerns with public attitudes towards people living with HIV (Mean: 7.7, SD: 2.57), internalized stigma (Mean: 6.45, SD: 2.76), and personalised stigma (Mean: 6.17, SD: 2.80). Using the cut off of > 7.5, most (78%) scored high on concerns with sharing HIV status and about one half (54%) scored high on concerns with public attitudes towards people living with HIV. High scores on personalised stigma and internalized stigma were reported by around one third of the participants respectively.

There were mutually statistically significant correlations between the four HIV-related stigma manifestations. The strongest correlations were between concerns with public attitudes towards people living with HIV and concerns with sharing HIV status (*r* = 0.54, *p* < 0.001) and concerns with public attitudes towards people living with HIV and internalized stigma (*r* = 0.50, *p* < 0.001).

### Bivariable one-way analysis

The bivariable one-way analyses of factors associated with the four HIV-related stigma manifestations are shown in Table 4.

**Table 4:** Bivariate one-way analyses of variance predicting each of the four HIV-related stigma manifestations

	Personalised stigma	Concerns with public attitudes	Concerns with sharing HIV status	Internalized stigma
	*N* = 1 004	*N* = 1 037	*N* = 1 060	*N* = 1 060
	F	F	F	F
**Demographic characteristics and clinical HIV factors**				
Gender	5.382**	11.419***	2.680*	3.693*
Education level	1.202	0.942	3.502**	0.299
Occupation	2.506*	5.176***	11.413***	1.232
Income	4.901***	0.615	4.649***	1.990
Residential status	2.821*	0.039	2.807*	2.528*
Sexual orientation	12.296***	12.224***	2.686*	5.065***
Relationship status	6.019**	0.126	1.870	5.101**
Drug use during the past six months	3.516*	6.023***	17.248***	1.861
Self-reported route of transmission	4.574***	3.061**	6.502***	1.203
Time since HIV diagnosis	3.677**	7.577***	4.393**	7.707***
**Distress and ART adherence**				
Feelings of hopelessness	24.823***	14.036***	5.817***	39.232***
Taking medication for sleeping problems	4.478*	1.886	0.249	9.240***
Impact of HIV on sex life	6.067***	2.489	6.293***	15.494***
Fear of being reported to the authorities	12.276***	11.359***	18.794***	23.632***
Fear of transmitting HIV to others	9.637***	6.306***	11.753***	21.478***
Wish to have children in the future	3.095**	3.944**	1.636	7.828***
Missed ART doses in the past week	6.176**	0.417	1.110	4.945**
**Available emotional HIV-related support**				
Emotional support from partner, family, friends and others	20.371***	11.047***	15.777***	14.072***
Trust in HIV physician	2.179	1.620	0.478	3.084*
Trust in HIV counsellor	2.871*	4.125**	0.175	1.939

*p < 0.05; **p < 0.01; ***p < 0.001

The following variables were non-significant: Age; Country/region of origin; Permit for Swedish residency; Having children; CD4 cells count; Currently experiencing HIV physical symptoms; ART treatment; ART side effects; Concurrent treatment for hepatitis C; Medication for worry/anxiety; Medication for depression; Medication for neuropsychiatric condition; Trust in HIV nurse

Income, sexual orientation and relationship status were excluded from multivariate analysis due to collinearity

### Multivariable analysis

Table 5 presents results from the multivariable hierarchical linear regression analyses of the four HIV-related stigma manifestations. Between 23 and 31% of the variance of the HIV-related stigma manifestations were explained by the same pattern of associated factors including female gender, shorter time since HIV diagnosis, feelings of hopelessness, non-sharing HIV status, and lack of available emotional HIV-related support. In addition, fear of being reported to authorities by a sexual partner associated significantly with higher scores on all the stigma manifestations except the personalised stigma. Data also showed significant associations between being a man who acquired HIV through sex with a woman and higher scores of the personalised, concern with public attitudes toward people living with HIV, and concerns of sharing HIV status concerns.

**Table 5:** Multivariate hierarchical linear regressions predicting each of the four HIV-related stigma manifestations among study participants

	Personalised stigma *N* = 1 004	Concerns with public attitudes *N* = 1 037	Concerns with sharing HIV status *N* = 1 060	Internalized stigma *N* = 1 060
**Demographic characteristics and clinical HIV factors**				
R^2^, *p* value	0.074***	0.101***	0.122***	0.052***
	β_s_	βs	βs	βs
*Gender*				
Male	Ref	Ref	Ref	Ref
Female	0.105**	0.172***	0.119***	0.088**
Other	-0.011	0.038	0.000	-0.037
Missing	-0.016	0.033	-0.011	-0.011
*Occupation*				
Working	Ref	Ref	Ref	
Not on the job market	-0.019	-0.053	-0.100***	
Long-term sick leave/ retired	-0.031	-0.118***	-0.148***	
Student	0.005	-0.029	-0.015	
Missing	-0.006	-0.113***	-0.051	
*Residential status*				
Live with partner/family	Ref		Ref	Ref
Share accommodation	-0.016		-0.086**	-0.025
Live alone	-0.068		-0.112***	-0.066
Have no home	0.041		-0.034	0.004
Missing	-0.030		-0.055	0.021
*Drug use during the past six months*				
No	Ref	Ref	Ref	
Non-injecting drug use	-0.058	-0.048	-0.046	
Injecting drug use	-0.010	-0.049	-0.143***	
Missing	0.048	0.058	-0.004	
*Self-reported route of HIV transmission*				
Through sexual contact with a man	Ref	Ref	Ref	
Through sexual contact with a woman	0.094**	0.100**	0.082**	
Through infected needles/syringes	-0.009	-0.001	-0.041	
Through blood/blood products	0.024	0.030	-0.037	
Through perinatal transmission	-0.007	-0.011	-0.009	
Other	0.076**	0.025	0.010	
Missing	0.053	0.059	-0.052	
*Time since HIV diagnosis*				
6 to 12 months	0.056	-0.010	0.035	0.052
1 to 5 years	0.090**	0.065	0.085**	0.129***
5 to 10 years	0.052	-0.040	-0.029	0.033
10 to 20 years	Ref	Ref	Ref	Ref
More than 20 years	0.026	-0.095**	-0.026	-0.051
Missing	0.021	0.037	0.034	0.028
**Distress and ART adherence**				
**Change in R**^**2**^, ***p*** **value**	0.126***	0.091***	0.093***	0.227***
*Feelings of hopelessness*				
Absence	Ref	Ref	Ref	Ref
Mild	0.088**	0.088**	0.054	0.097***
Moderate	0.129***	0.100***	0.050	0.185***
Severe	0.224***	0.210***	0.158***	0.258***
Missing	0.045	0.041	0.014	0.069**
*Taking medication for sleeping problems*				
No	Ref			Ref
Yes	-0.003			0.035
Missing	0.051			0.070**
*Impact of HIV on sex life*				
Negative	Ref		Ref	Ref
Positive	0.085**		-0.063	-0.079**
No change	-0.048		-0.107***	-0.135***
Missing	-0.019		-0.059	-0.059
*Fear of being reported to the authorities*				
No	Ref	Ref	Ref	Ref
Yes	0.063	0.083**	0.086**	0.091**
Missing	0.068	0.059	0.082**	0.105***
*Fear of transmitting HIV to others*				
No	-0.078	-0.062	-0.096**	-0.129***
Yes	Ref	Ref	Ref	Ref
No sex	-0.028	-0.067	-0.037	-0.076**
Missing	-0.028	-0.050	-0.023	-0.117***
*Wish to have children in the future*				
No	Ref	Ref		Ref
Yes, it is very important	0.038	0.061		0.140***
Yes, but it is not so important	0.031	0.055		0.055
Yes, but did not know people living with HIV could have children	-0.002	0.021		0.025
Do not know	0.048	0.030		0.074**
It is too late	0.040	0.019		0.011
Missing	-0.020	0.023		-0.029
*Missed doses of ART in the past week*				
No	Ref			Ref
One or more	0.014			0.029
Missing	0.021			0.001
**Available emotional HIV-related support**				
**Change in R**^**2**^, ***p*** **value**	0.06***	0.039***	0.049***	0.031***
*Emotional support from partner, family, friends and others*				
Always	Ref	Ref	Ref	Ref
Often	0.062	0.022	-0.003	0.072
Sometimes	0.152***	0.083	0.102**	0.138***
Rarely	0.120***	0.088**	0.092**	0.112***
Never	0.110***	0.082**	0.072	0.079**
I have not told anyone about my HIV	0.287***	0.203***	0.251***	0.178***
Missing	0.090**	0.045	0.112**	0.091**
*Trust in HIV physician*				
Completely				Ref
Partly				-0.026
No				-0.089***
Missing				-0.018
*Trust in HIV counsellor*				
Completely	Ref	Ref		
Partly	0.027	0.013		
No	0.059	0.102***		
Missing	0.070	0.026		
Final model R^2^, *p* value	0.261, < 0.001	0.231, < 0.001	0.264, < 0.001	0.311, < 0.001

*p < 0.05; **p < 0.01; ***p < 0.001

βs = standardized beta coefficients

The variable Education level was non-significant

## Discussion

To our knowledge, this is the first study to examine the prevalence of four HIV-related stigma manifestations and their associated individual characteristics in people living with HIV in Sweden. In accordance with earlier international studies [52, 53], a majority reported high levels of concerns with sharing HIV status indicating caution in sharing one’s HIV status due to fear/worries to be rejected, abandoned and/or poorly treated by others. About half of the participants reported high concerns with public attitudes towards people living with HIV. The relatively low number of participants reporting high level of internalized stigma (34%) are in line with a Danish study among women living with HIV [54] but in contrast to U.S. data, where 8 in 10 people living with HIV reported internalized stigma [55]. Types of measurement and criteria of level of HIV-related stigma may account in part for differences across studies. One third of the participants had experienced rejection after HIV disclosure, i.e. experienced personalised stigma. Hedge et al. [56]. suggest that personalised stigma has diminished but “participants were unclear whether this is related to the increasing acceptance and/or the increased invisibility of people living with HIV” ([56], p. 9). Stutterheim et al. [57] conclude that stigma from friends, family, acquaintances, at work, and in the financial services sector had reduced in recent years in the Netherlands. But stigma in health care has increased and stigma in the LGBTQI + community remained relatively unchanged.

As alluded in previous research, the correlation data clearly indicated that people living with HIV may perceive more than one manifestation of HIV-related stigma, The strongest correlations were between concerns with public attitudes towards people living with HIV and concerns with sharing HIV status and concerns with public attitudes towards people living with HIV and internalized stigma respectively.

Unanticipated, the results demonstrated across all four HIV-related stigma manifestations mainly the same pattern of associated factors linked to the three layers: Female gender, shorter time since HIV diagnosis (the first layer, demographic characteristics and clinical HIV factors), hopelessness (the second layer, distress) and lack of available emotional HIV-related support from loved ones and friends (the third layer, available emotional HIV-related support).

### Female gender

Women are more often stigmatized than men for having HIV [17, 42, 58]. It demonstrates that gendered stigmatization exists in our society, which was also reflected in the present study. Women were more likely than men to have experienced personal stigmatization, to report higher levels of anticipated as well as internalized stigma. Over two thirds of the participating women had non-Swedish origin, but in contrast to other studies, we did not find associations between female gender and non-Swedish origin [59].

In agreement with findings of Logie et al. [60], adding available emotional HIV-related support in the multivariable analysis did not lessen the association between female gender and perception of HIV-related stigma.

### Shorter time since HIV diagnosis

In accordance with other studies, participants who got their HIV diagnosis 1–5 years prior to data collection were more vulnerable to perceive high levels of personalised stigma, concerns of sharing HIV status and internalized stigma than people living with HIV diagnosed 10–20 years prior to data collection [61]. It could be speculated that the finding reflects an ongoing adaptation process in which people living with HIV need time to psychologically and socially navigate and cope with and gain perspective on the aftermath of an HIV diagnosis. On the other hand, people living with HIV who were notified about their HIV diagnosis more than 20 years prior to data collection reported lower levels of concerns about public attitudes towards people living with HIV. This finding is supported by extant research that perceived stigma on average decreases over time as a result of adjusting to HIV status [62]. It could be argued that individuals who have been living with HIV for a longer time have developed strategies to cope with negative attitudes towards people living with HIV. Another explanation may be that one becomes less concerned, sensitive to, or fearful of social stigma over time [63]. Societal attitudes towards HIV have also changed in Sweden over the years into a less negative direction [64].

### Hopelessness

As well as HIV-related stigma is directly or indirectly associated with psychological distress [45, 53, 65–67], psychological distress may also increase attention to and affect perception of HIV-related stigma in its different appearances. In this study, distress in terms of feelings of hopelessness was the strongest correlate of all four HIV-related stigma manifestations, in particular internalized stigma. Hopelessness is indicated by feelings of low personal control, expectations of negative outcomes and feelings of helplessness to change those expectations [45, 49]. The individual may thus presuppose to be negatively treated by others because of HIV status. A substantial minority of the participants had also experienced discriminatory behaviours due to their HIV status, which in turn may reinforce feelings of hopelessness. Moreover, hopelessness contributed to internalized stigma. It could be speculated that due to feelings of no personal control, there is no intrapersonal protection towards intrusion of societal negative HIV attitudes into the individual’s mindset.

### Non-sharing HIV status

Sharing HIV status is a way to receive emotional HIV-related support, but it also allows for the possibility of stigmatisation [42]. In this study, 17% of the participants had not shared their HIV status to anyone outside the healthcare system. One possible interpretation may be that some people living with HIV worry about being badly treated upon sharing HIV status and therefore choose self-imposed social isolation to not risk revealing one’s HIV status. Among others, another potential reason for non-sharing HIV status might be that one considers one’s HIV status as a private matter, not of concern for anyone else [68]. It is also conceivable that individuals who had not shared their HIV status may had incorporated societal negative attitudes. In that way one may become one’s own “persecutor” [69].

### Lack of available emotional HIV-related support

Findings indicated that lack of available emotional HIV-related support correlated with higher scores of all four HIV-related stigma manifestations, an association supported by prior research [37]. It is quite likely that experiences of unmet support needs will lead to lack of trust in others for support as reflected in sharing HIV status concerns but also in concerns about public attitudes towards people living with HIV. Among HIV-positive people scoring high on internalized stigma, it is plausible that limited emotional support may be inferred as a confirmation of feelings of not being good enough to deserve support and care. As a consequence, one may withdraw from social relationships in an attempt to minimize risk of rejection by and disappointment in others. It may bring about social isolation and hence lessened chances for emotional social support.

As our findings are based on data collected 10 years ago, a pertinent question is whether experiences of HIV-related stigma in Sweden have changed. The Swedish biomedical situation in terms of ART coverage and viral suppression is the same today as it was in 2014, when the current study was conducted, i.e. 98% ART coverage with over 95% reaching successful viral suppression [1]. The Public Health Agency of Sweden performed in 2021 a web-based study among people living with HIV (*n* = 296) [70]. The aim of the study was to monitor the health, living conditions, and quality of life among people living with HIV in Sweden. HIV-related stigma was measured by the same instrument as the one in our study. The results supported our findings in that most of the participants experienced high levels of anticipatory stigma, whereas internalized stigma and personalised stigma were less common (70). Another Swedish study, conducted in 2020–2021, showed almost identical mean scores of the four HIV-related stigma manifestations as ours among people living with HIV and who had experienced a COVID-19 event [71]. These results suggest that our findings related to prevalence of HIV-related stigma from 2014 are still valid and relevant in Sweden and supported by international literature that anticipated stigma still creates challenging and difficult issues for people living with HIV [33, 56, 72]. In other words, HIV-related stigma prevails despite the fact that treated HIV is not severe and not sexually transmissible.

As there are no Swedish studies examining associated factors of HIV-related stigma manifestations, we do not know whether the correlates identified in this study are still legitimate. But our findings related to associate factors are, 10 years later, supported by current international HIV-related stigma literature. The difference between the present study and other studies is that we identified mainly the same factors associated with all four HIV-related stigma manifestations, whereas most international studies have primarily focused on contributors of total HIV-related stigma or only on one or two manifestations.

From a clinical perspective, it is essential that healthcare personnel providing services to people living with HIV are aware and attentive to potential vulnerability factors. People living with HIV suffering from hopelessness should be offered relevant psychosocial treatment including referrals to mental health services when needed. Access to social support and feeling supported are important buffers to stressors such as HIV-related stigma [73]. HIV peer support constitutes a vital resource in the support of stigma and illness burden [74]. Information about HIV peer support should be shared and discussed with clients and referral made available. Moreover, as anticipated stigma was the most dominating HIV-related stigma feature, it underlines the imperative necessity to support people living with HIV to increase resilience to HIV-related stigma in its different forms.

The present study highlights the salience of developing individual level stigma reduction interventions of anticipated stigma, as it has significant associations with concealment of HIV. Moreover, interventions focusing on individual factors, such as hopelessness, shorter time since HIV diagnosis, interpersonal factors such as non-sharing HIV status and lack of emotional HIV-related support may contribute towards reduction of HIV stigma among people living with HIV. Women are of concern as they reported higher levels on all four HIV-related stigma manifestations than men did. Thus, tailored efforts to reduce stigma for women living with HIV should be developed.

Stigma-reduction interventions also need to address negative HIV-related attitudes and beliefs among the general population, health care and judiciary. Information-based interventions are important to increase HIV-related intellectual and emotional knowledge but also to tackle outdated HIV beliefs and stigmatizing language. As individual stigma-reductions interventions cannot be performed isolated from HIV-related stigma and discrimination in society, a key challenge is to intensify anti-stigma interventions also on the societal level.

### Limitations and strengths

As the data was collected cross-sectionally, conclusions about causal effects cannot be drawn. For example, we are unable to determine any directionality regarding hopelessness and HIV-related stigma. Does hopelessness contribute to stigma or is hopelessness an outcome of HIV-related stigma?

Data were gathered through a self-report questionnaire, so there may have been inaccuracies in reporting HIV-related clinical information. Although the survey was anonymous, response bias cannot be excluded. Furthermore, there is no information about reasons for not participating in the study. Additionally, the sample included a smaller proportion of female gender compared to the national demographics of people living with HIV (29% vs. 38%) and a higher proportion of Swedish-born participants than in the population of people living with HIV in Sweden (55% vs. 36%), which may affect the generalizability of the findings. Another limitation is that the meaning of statistical significance of a ‘missing’ response on a given variable cannot be further understood or explained as the response categories were a mixture of ‘do not know’, ‘do not want to answer’ or a missing response.

The four models all showed R^2^ values of medium effect size, with 23–31% of the variance explained. This indicates that a significant share of the variance in the HIV-related stigma manifestations were explained by the independent variables, although parts of the variance remain unexplained. The unexplained variance could for example be related to effects of intersectional stigma of ethnicity/sexual orientation and or structural stigma within health care facilities. However, the original survey *Living with HIV in Sweden* did not include measures on neither intersectional nor structural stigma. Regarding magnitude of the βs values of the independent variables from the final step of the respective multivariate hierarchical linear regression, severe feelings of hopelessness and not having shared one´s HIV status with anyone showed medium effect sizes. The remaining variables with statistically significant contribution to the respective multivariate model showed βs values of weak effect sizes.

Regarding the imputation scheme applied for the four HIV-related stigma manifestations, for those with two out of three missing answers on a manifestation, the two missing values were imputed with the individual’s single response on the remaining item. This procedure may risk having skewed the data. However, it was only a few respondents that had 2 out of 3 missing answers: eight for internalized stigma, 13 for concerns with public attitudes, 14 for concerns of sharing HIV status and 29 for personalised stigma. Excluding respondents with 2 out of 3 missing answers gave no or minor changes on the mean and SD on the respective scale: internalized stigma: mean 6.45 (sd 2.76) for all vs. 6.43 (2.75) when those with 2 out of 3 responses missing excluded; concerns with public attitudes towards people living with HIV: 7.72 (2.57) vs. 7.72 (2.56); concerns with sharing HIV status: 9.18 (2.58) vs. 9.18 (2.58) and for internalized stigma: 6.17 (2.80) vs. 6.11 (2.76). We therefore conclude that the imputation procedure used resulted in minor impact on the results.

The key strength of the present study is its nationwide, multicultural and a fairly representative sample including approximately 17% of people living with HIV in Sweden. Another strength is the engagement of the HIV community, non-governmental organisations and clinicians in the development and dissemination of the survey. Given the similarities of our prevalence and associated factors findings with international research, we suggest they could be transferable at least to the Nordic countries and Western Europe.

## Conclusions

The most dominating stigma feature in this nationally representative study of people living with HIV in Sweden was anticipation of stigma. Female gender, shorter time since HIV diagnosis, feelings of hopelessness, non-sharing HIV status, and lack of available emotional HIV-related support constituted potential vulnerability factors of the four HIV-related stigma manifestations. Our findings highlight the vital necessity to support people living with HIV to increase their resilience to stigma in its different forms. Exploring associated factors of HIV-related stigma manifestations may give an indication of what circumstances may increase the risk of stigma burden and factors amenable to targeted interventions. As individual stigma-reductions interventions cannot be performed isolated from HIV-related stigma and discrimination in society, a key challenge is to intensify anti-stigma interventions also on the societal level.

## Data Availability

All data analysed during this study is included in this article.

## Abbreviations

HIV: Human immunodeficiency virus
ART: Antiretroviral therapy
CD4: T lymphocytes or helper T cells
